# AGE-BASED STEREOTYPE THREAT AND INTENTION TO WORK BEYOND RETIREMENT AGE: TESTING THE INDIRECT EFFECT THROUGH STRESS

**DOI:** 10.13075/ijomeh.1896.02627

**Published:** 2025

**Authors:** Zofia Mockałło, Sylwia Bedyńska, Dorota Żołnierczyk-Zreda

**Affiliations:** 1 Central Institute for Labour Protection – National Research Institute, Laboratory of Psychology and Sociology of Work, Department of Ergonomics, Warsaw, Poland; 2 SWPS University, Center for Research on Social Relations, Institute of Psychology, Warsaw, Poland

**Keywords:** stress, age, older employees, stereotype threat, intention to work beyond retirement age, age-based stereotype threat

## Abstract

**Objectives:**

Stereotype threat arises when an individual worries about the possibility of confirming or being perceived through the lens of a negative stereotype about one's group. Previous research has shown that stereotype threat at work is related to higher stress appraisal and an increased intention to quit among older employees. The present study extends these investigations by examining the links between stereotype threat and post-retirement work intention, as well as indirect effects through stress.

**Material and Methods:**

The level of age-related stereotype threat, stress, and intention to continue working beyond the retirement age were assessed using self-reported measures in a cross-sectional study among working adults aged ≥50 years (N = 1007). The average age of participants was 56.3 years (standard deviation 4.2 years). The sample included both men and women, with diverse education levels and job types (mental, physical, and mixed work), and was drawn from various sectors, including public and private organizations.

**Results:**

The authors' findings indicated that a higher level of stereotype threat was linked to higher level of stress and willingness to resign from work after reaching retirement age in aging workers. Stress level partly transmitted the relationship between stereotype threat and intention to resign from work after reaching the retirement age.

**Conclusions:**

It can be concluded that stress is a significant variable constituting the indirect effect of age-based stereotype threat at work on post-retirement work intention, when physical health is statistically controlled. These results are discussed in the context of recent studies on age-related stereotype threat in occupational settings.

## Highlights

- Stereotype threat is linked to higher stress and lower intent to continue working.
- Stress mediates the effect of stereotype threat on the intention to quit when retired.
- Addressing stereotype threat can improve mental health and retention of aging workers.

## INTRODUCTION

Although the role of stereotype threat in education has been empirically examined for 30 years, few studies have investigated the role of stereotype threat in occupational context, particularly concerning the relationship between stereotype threat, perceived stress, and intention to work beyond retirement age in older adults samples. The present study draws on previous research and focuses on the role of stress as a potential mediator of the association between age-related stereotype threat and willingness to work after reaching retirement age. Below the authors justify these predictions within the theoretical process-oriented framework of the transactional model of stress and coping, developed by Lazarus and Folkman [1].

The transactional model of stress and coping [1], posits that stress arises from complex interactions between individuals and their environment, when individuals evaluate the situation as a threat or harm in the process of primary appraisal. This cognitive process evokes distress, provoking negative emotions that initiate different coping strategies. When a situation is appraised as stressful, secondary appraisal is initiated and individuals assess their ability to cope with the situation. Coping strategies are then implemented and broadly classified into 2 categories:

– problem-focused coping, aimed at directly managing the stressor,
– emotion-focused, aimed at regulating negative emotion.

Following the transactional model of stress and coping, resignation from the current workplace [2] or from employment in general among older adults may be understood as a consequence of ineffective coping strategies. In contrast, conservation of resources (COR) theory [3] proposes that the intention to resign is driven by the motivation to protect valuable resources – such as health, well-being, or positive self-image – when these resources are threatened in the present or anticipated to be lost in the future. Within this framework, stereotype threat may serve as a signal of potential resource depletion, including reduced social support, a diminished sense of self-worth, or the possible loss of one's job position. Thus, although the 2 theories differ fundamentally in their explanation of the stress response, they converge in predicting similar outcomes: the intention to resign from work or actual turnover behavior.

Studies predicting willingness to work beyond retirement age are of great importance as the number of older people among all employees has been steadily increasing in the recent years [4]. Moreover, due to demographic changes, governments are interested in stimulating labor force participation among older workers. Additionally, growing research has demonstrated that working past retirement age may be beneficial not only for social systems but also for older employees by offering mental and social stimulation [5]. As policy efforts increasingly promote prolonged working lives, there is a growing need to better understand the psychological factors that may either support or hinder older workers' intention to remain employed.

Post-retirement work intentions may be driven by a wide range of psychological, social and health factors [6]. On the one hand, post-retirement work is perceived as an adaptive strategy to cope with the possible loss of financial and social benefits of the employment. Regarding work functions, Bratun et al. [7] identified possible benefits for older individuals, including achievement, positive relationships, helping others, and enjoying work. On the other hand, the decision to stop working after reaching retirement age may be motivated by desire to avoid negative elements of work environment that elicit stress [8]. For instance, person-environment fit theory explains decision-making process about working after reaching retirement age in the context of the congruence between older adults' needs and important workplace features [9]. Feldman and Beehr [10] noted that older employees may perceive incongruence due to decline in their physical or cognitive abilities, but incongruence may also arise from working environment, such as prevailing age stereotypes.

Negative stereotypes about aging are widespread, and older workers are frequently negatively stereotyped as lower-performing, less able to learn, resistant to change, and more costly [11]. These stereotypes have a negative influence on work effectiveness and well-being of older employees. One possible mechanism is stereotype threat, defined as “a threat of possibly being judged and treated stereotypically, or of possibly self-fulfilling such a stereotype” [12].

Numerous experimental studies have confirmed that stereotype threat referring to “old and senile” stereotype of older adults significantly affects cognitive performance, including decline in memory recall and working memory [13]. In occupational studies, stereotype threat experienced by older employees is associated with various negative job-related outcomes, such as lower job satisfaction, overall well-being, and work self-efficacy [14,15]. Older adults with higher levels of stereotype threat also demonstrate lower job commitment and greater intention to resign from work or retire as soon as possible [16]. Generally, this analysis confirmed that stereotype threat is substantially related to all work-related outcomes, with the strongest relationships observed for turnover intentions, identity separation, and lack of positive affect. More importantly, the negative associations between stereotype threat and career aspirations, work satisfaction, and engagement were stronger for older adults than for female workers. However, among >40 studies included in a recent meta-analysis [15] on the effects of stereotype threat at work, only 13 explored stereotype threat in older adults samples. Therefore, since the number of studies examining the relationship between stereotype threat and intention to resign is very limited, the mechanism linking consequences of stereotype threat to intention to quit in older adults sample in the workplace remains unclear. In the study, the authors address this issue by examining the role of stress in the intention to work after reaching retirement age among older participants.

Several psychological mechanisms explaining the negative outcomes of stereotype threat at work have been recently proposed [17] and examined [16]. Studies showing that stereotype threat experienced at work leads to a higher level of stress are particularly relevant to the current study. To the authors' knowledge, only 2 studies have examined the role of stress in the stereotype threat mechanism.

In the first study, von Hippel et al. [16] explored the negative effects of daily stereotype threat incidents at work among older employees. Contrary to the predictions, the study provided evidence that reduced challenge appraisal rather than increased hindrance appraisal, was an important mediator of the relationship between stereotype threat and work outcomes. Older employees who experienced stereotype threat more frequently, were less likely to appraise stressful situations at work as challenges and reported lower job satisfaction, job engagement, and workplace well-being. In the second study, Coulon et al. [18] found that experiencing more daily stereotype threat events was related to a higher number of workplace stress events, although this relationship was relatively weak. However, this exploratory analysis only provided evidence of the relationship between stereotype threat and stress, with no reference to intention to resign form working in the present organization as a way of coping with stress in the workplace.

### The current study

Based on the transactional model of stress and coping [1], COR theory [3], and empirical research on older adult employees, the authors assume that as negative stereotypes about older employees are widespread, older adults may experience higher levels of stereotype threat at the workplace, even when organizational culture in their current organization fosters inclusive behaviors that challenge these stereotypes. This worry of being perceived through negative stereotypes, named stereotype threat, may be perceived as a significant workplace stressor and signal of potential resource depletion, leading to higher levels of perceived stress.

Following these premises, this study had 2 primary objectives. First, the authors aimed to replicate previous research by examining the relationship between self-reported stereotype threat and the intention to work past retirement age. Given that intention to work after reaching retirement age may be strongly related to physical health, the authors also decided to control for health level as a covariate. Second, this study sought to clarify the underlying stress-related mechanism through which stereotype threat relates to the intention to work beyond retirement age. Therefore, in relation to the research aim and the employed theoretical framework, the following hypotheses were formulated:

– H1: The level of age-related stereotype threat is positively associated with stress and negatively with intention to work beyond the retirement age;
– H2: Stress is a mediator of the relationship between age-related stereotype threat and intention to work beyond the retirement age.

## MATERIAL AND METHODS

### Participants and procedure

The study group consisted of 1007 participants (56.7% female). The age of participants age was mean (M) ± standard deviation (SD) 56.27±4.17 years, and their job tenure was M±SD 32.42±6.68 years. The vast majority of the group was married or in a partnership, and had completed upper secondary or higher education. They were employed in a wide range of public and private organizations within the service and industrial sectors. Overall, 42.6% of the group considered their job to be mainly mentally demanding, while 37.3% described it as mainly physically demanding. Most participants (76.3%) held a permanent (indefinite duration) contract, followed by those with fixed-term (definite duration) contracts. A detailed description of the sample is presented in Table 1.

**Table 1.** Sociodemographic characteristics of participants in the study among employees from various organizations, Poland, 2015

Variable	Participants (N = 1007)	
	n	%
Gender		
female	571	56.7
male	420	41.7
Marital status		
single	35	3.5
married/partnered	808	80.3
separated/divorced	100	9.9
widowed	52	5.2
Age		
50–55 years	485	48.2
56–60 years	374	37.1
61–65 years	124	12.3
≥66 years	24	2.4
Highest educational level		
primary education	23	2.3
lower secondary education	198	19.7
upper secondary education	331	32.8
post-secondary non-tertiary education	91	9.0
bachelor's or equivalent level	38	3.8
master's or equivalent level	229	22.7
doctoral or equivalent level	33	3.3
Type of work		
mainly mental demands	429	42.6
mainly physical demands	376	37.3
both mental and physical demands	187	18.6
Type of job contract		
civil law contract	90	8.9
temporary work	19	1.9
definite duration contract	95	9.4
indefinite duration contract	768	76.3

The sample was selected using convenience sampling. Inclusion criteria comprised:

– age >49 years,
– current employment (permanent or fixed-term contract),
– fluency in Polish sufficient to complete a self-report questionnaire,
– representation of both genders,
– inclusion of employees from a variety of sectors and job types (mental, physical, and mixed work).

This study is based on data collected through a cross-sectional paper-and-pencil questionnaire survey carried out by an external research agency across 95 organizations operating in 25 locations in Poland (Europe) in 2015. After obtaining permission from the participating organizations, interviewers invited employees who met the inclusion criteria to participate. Interviewers informed potential participants about the study's purpose, the voluntary nature of participation, the assurance of anonymity, and the right to withdraw at any time. Employees who agreed to participate received a set of questionnaires to be completed at their convenience. Completed questionnaires were sealed in pre-provided envelopes and collected by the interviewer on a predetermined date.

### Measures

#### Stereotype threat

Stereotype threat was measured using the 5-item scale developed by von Hippel et al. [19]. A sample item is: “Some of my colleagues feel that I have less to contribute because of my age”. Participants indicated the extent to which they experience age-based stereotype threat at work on a 7-point Likert scale ranging from 1 (absolutely disagree) to 7 (absolutely agree). Despite consisting of only 5 items, the scale demonstrated high reliability, with Cronbach's α = 0.90. Since stereotype threat was represented in the mediational model as a latent variable loaded by the scale items, the assumed 1-factor structure was assessed using confirmatory factor analysis (CFA) in Mplus 8.2 (Muthén & Muthén, Los Angeles, USA) with maximum likelihood robust (MLR) estimation due to non-normal item distributions. Modification indices from the preliminary model suggested adding covariance between items 4 and 5 to improve model fit. After this modification, the model demonstrated excellent fit: χ^2^ (4, N = 1006) = 416.22, p = 0.003, root mean square error of approximation (RMSEA) = 0.06, p = 0.33, 90% confidence intervals (CI): 0.03–0.08, comparative fit index (CFI) = 0.99, Tucker-Lewis index (TLI) = 0.98. Factor loadings ranged from 0.64 to 0.92.

#### Stress

Stress level was assessed using a subscale of the *Copenhagen Psychosocial Questionnaire* v. 2 (COPSOQ II) [20], validated in Polish [21]. Participants rated their stress level stress using 4 items on a 5-point Likert scale, ranging from 1 (all the time) to 5 (not at all). A sample item is: “How often have you had problems relaxing?”. Unlike the original COPSOQ II scale, where responses were coded with values 0–100 (i.e., 0, 25, 50, 75, and 100 for the 5 response categories), the authors coded participants' responses from 1 to 5.

This coding approach was adopted to meet structural equation modelling requirements concerning variance similarity across all observed variables in the model [22]. Similar to the approach applied to the stereotype threat measure, stress was modeled as a latent variable in the mediational model, with its unidimensional structure confirmed through CFA using Mplus 8.2 and MLR estimation due to non-normal item distributions. Modification indices of the preliminary model suggested adding covariance between items 3 and 4 to improve the model fit. After this modification the model demonstrated excellent fit: χ^2^ (1, N = 1003) = 3.49, p = 0.062, RMSEA = 0.05, p = 0.39, 90% CI: 0.001–0.111, CFI = 0.99, TLI = 0.99. Factor loadings ranged from 0.72 to 0.84. The stress subscale demonstrated high internal reliability (Cronbach's α = 0.87).

#### Intention to continue working beyond the retirement age

Intention to continue working beyond retirement age was assessed using a single item where participants indicated whether they intended to continue working after reaching retirement age. Participants described their intention using 3 options: “I am going to resign from work entirely,” “I am going to work part-time after being retired,” and “I am not going to retire.” The authors combined the latter 2 options to create a dichotomous indicator where 0 represented respondents who reported intention to quit work upon retirement, and 1 – those who reported intention to continue working beyond the retirement age (either part-time or full-time).

#### Health status

Health status was measured using the number of diagnosed diseases from the third dimension of the *Work Ability Index* [23], which enumerates 14 disease groups. Respondents indicated whether they suffered from a given disease group and whether these conditions were diagnosed by a physician. The physician-diagnosed diseases were summed to form a composite index.

### Ethics

The research was conducted in accordance with the Polish National Academy of Sciences code of ethics [24] and the Helsinki Declaration, as revised in 2013 [25].

Participants were fully informed about the purpose and procedure of the study before taking part, and participation was entirely voluntary. They were informed of their right to withdraw at any time. The study was conducted anonymously: questionnaires were distributed in envelopes and returned in sealed envelopes. No personal data were collected, and only basic sociodemographic information was obtained, ensuring privacy and preventing identification of individuals. The research instruments were carefully selected to avoid imposing substantial cognitive or time burdens on participants. Moreover, participants could express interest in being informed about the study's results. Overall, the study design prioritized participants' dignity, rights, and well-being, while minimizing any potential risks.

### Statistical analyses

Structural equation modeling (SEM) analyses were conducted using Mplus 8.3 [26]. The authors constructed a model with stereotype threat as the predictor, stress as the mediator, and intention to continue working beyond the retirement age as the dependent variable, while controlling for health status. Stereotype threat and work stress were represented as latent variables constructed from their respective scale items. None of the variables used in the analysis were normally distributed (Table 2). Due to the dichotomous dependent variable and non-normally distributed variables, the authors used weighted least squares mean and variance-adjusted (WLSMV) estimation methods to obtain model fit statistics. Indirect effects were evaluated using the INDIRECT function in Mplus with 90% CI, with indirect effects considered significant if the CI did not include 0 [22].

**Table 2.** Descriptive statistics and correlation coefficients for the model linking stereotype threat to intention to work beyond the retirement age through stress in the study among 1007 employees from various organizations, Poland, 2015

Variable	M	SD	Z	Pearson's r									
				1	2	3	4	5	6	7	8	9	10
1. Number of diseases	1.22	1.46	0.23**										
2. Stereotype threat – item 1	2.30	1.40	0.24**	–0.01									
3. Stereotype threat – item 2	2.30	1.41	0.25**	0.09*	0.82**								
4. Stereotype threat – item 3	2.38	1.40	0.24**	0.10*	0.78**	0.82**							
5. Stereotype threat – item 4	3.04	1.77	0.20**	0.24**	0.56**	0.59**	0.63**						
6. Stereotype threat – item 5	2.88	1.40	0.20**	0.17**	0.57**	0.57**	0.58**	0.65**					
7. Stress – item 1	2.48	0.85	0.23**	0.18**	0.21**	0.20**	0.21**	0.22**	0.27**				
8. Stress – item 2	2.58	0.88	0.23**	0.14**	0.19**	0.16**	0.19**	0.23**	0.25**	0.58**			
9. Stress – item 3	2.56	0.89	0.23**	0.19**	0.20**	0.21**	0.20**	0.23**	0.26**	0.55**	0.67**		
10. Stress – item 4	2.52	1.46	0.23**	0.19**	0.13**	0.16**	0.16**	0.21**	0.23**	0.52**	0.59**	0.71**	
11. Working beyond the retirement age (0 – no, 1 – yes)	–	–	–	0.01	–0.17**	–0.14*	–0.18**	–0.13*	–0.12*	–0.20**	–0.19**	–0.13*	–0.14*

Z – Kolmogorov-Smirnov normality test, N = 1007.

* p < 0.01;

** p < 0.001.

The structural model was evaluated using robust χ^2^ statistic and the RMSEA, the standardized root mean square residual (SRMR), CFI, and the TLI, as recommended by Kline [22]. The authors used widely recommended cutoff values indicative of adequate model fit: RMSEA and SRMR <0.06, and CFI and TLI >0.90 [27]. As presented in the literature, MLR and WLSMV estimation methods demonstrate comparable performance with ordinal and non-normally distributed data [22]. Therefore, to provide interpretation of relationships between predictors and the dependent variable in terms of odds ratio (OR), the authors also estimated their model using the MLR approach.

## RESULTS

### Descriptive statistics

The correlations between the variables included in the model linking stereotype threat to the intention to work beyond the retirement age with indirect effect through stress, along with relevant descriptive statistics, are presented in Table 2.

Overall, the level of stereotype threat was moderate, with its average value around the midpoint of the scale. Similarly, the level of stress was moderate, with all items showing mean values close to the center of the 5-point response scale. The number of diagnosed diseases was relatively low and demonstrated weak correlations with all measured variables.

### Relationship between stereotype threat and intention to work beyond retirement age, with an indirect effect through stress

The proposed model included a dichotomous variable, namely intention to work beyond retirement age, where willingness to work beyond the retirement age was coded as 1. The predictor was a latent variable assessing the level of stereotype threat, measured by 5 indicators (items). The authors also tested the indirect effect of stereotype threat through stress level, modeled as a latent variable with 4 indicators. Additionally, the authors used the number of diagnosed diseases as a covariate related to perceived stereotype threat, stress, and intention to work beyond retirement age.

The model including stress as a mediator demonstrated a good fit to the data. Although the overall test of fit was significant χ^2^(36) = 124.02, p < 0.001, the inspection of the fit values supported a relatively good model fit: CFI = 0.96, TLI = 0.93, SRMR = 0.03, RMSEA = 0.05, 90% CI: 0.04–0.06, p = 0.53.

**Figure 1.**
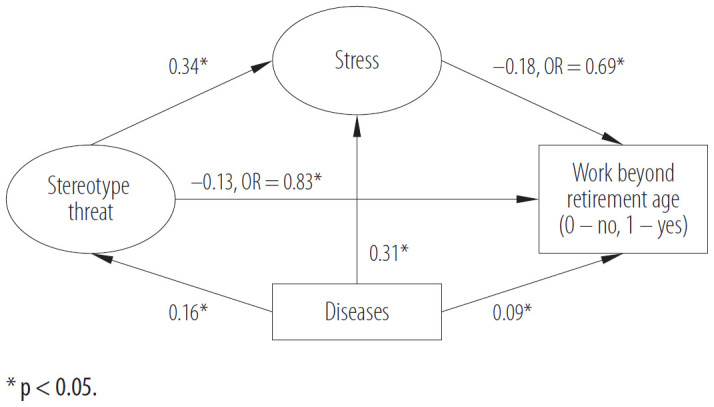
Model linking stereotype threat and intention to work beyond retirement age through stress, with number of diseases as a covariate, in the study among 1007 employees from various organizations, Poland, 2015

As predicted, stereotype threat was positively and moderately associated with stress, and stress at work was negatively related to the intention to work beyond retirement age (Figure 1). Therefore, a higher level of stereotype threat is related to higher stress and significantly related to a lower intention to work beyond retirement age. Unstandardized and standardized path coefficients, standard errors, critical values of Student's t-tests, and confidence intervals are presented in Table 3. The estimated path parameters of the structural model can also be described in terms of OR. Odds ratio for the relationship between stress and the intention to work beyond retirement age significantly differed from 1 with OR = 0.69, p < 0.001, 95% CI: 0.56–0.85. The OR for the relationship between stereotype threat and the intention to work beyond retirement age significantly differed from 1 with OR = 0.83, p = 0.001, 95% CI: 0.74–0.94.

**Table 3.** Path coefficients in the model linking stereotype threat to intention to continue work beyond the retirement age through stress, in the study among 1007 employees from various organizations, Poland, 2015

Path	B_a_	B_b_	SE	CR	p	95% CI
Stereotype threat → Stress	0.26	0.34	0.03	11.06	<0.001	0.20–0.32
Stereotype threat → Work beyond the retirement age	–0.14	–0.13	0.05	2.84	0.004	–0.24–(–0.04)
Stress → Work beyond the retirement age	–0.25	–0.18	0.05	3.70	<0.001	–0.39–(–0.12)
Diseases → Stereotype threat	0.10	0.16	0.03	4.87	<0.001	0.06–0.15
Diseases → Stress	0.15	0.31	0.03	10.71	<0.001	0.12–0.18
Diseases → Work beyond the retirement age	0.06	0.09	0.04	2.21	<0.001	0.01–0.12

B_a_ – unstandardized path coefficients; B_b_ – standardized path coefficients; CR – critical ratio; SE – standard error.

The statistics for the indirect effect from stereotype threat to the intention to work beyond retirement age indicated that this effect was significant effect = −0.06, p = 0.001, with 95% CI: −0.10–(–0.03). The direct effect of stereotype threat on the intention to work beyond retirement age was also significant effect = −0.13, p = 0.004, with 95% CI: −0.23–(−0.04).

## DISCUSSION

The present study significantly extends the recent research on the relationships between stereotype threat and willingness to work beyond retirement age in older adults by examining the mediational role of stress in this relationship. Based on the transactional theory of stress, the authors posited that stereotype threat situations in the workplace may evoke higher level of stress and consequently, which in turn may reduce employees' willingness to continue working beyond retirement age. This hypothesized relationship was further supported by the COR theory, proposing that stereotype threat may serve as a signal of potential resource depletion. From this perspective, the intention to withdraw from work can be interpreted as a protective strategy aimed at conserving valuable resources in the face of anticipated loss.

Indeed, the findings confirmed both of the authors' hypotheses. Older employees who reported higher levels of stereotype threat at work also reported higher levels of stress symptoms and were more likely to avoid working after reaching retirement age. Although the size of the indirect effect was moderate, it was significant and suggests that stress may transmit the effects of stereotype threat experienced at work into reduced willingness to continue working beyond the retirement age. These results significantly extend previous research on the relationship between stereotype threat and stress [18] by showing that stereotype threat may shape not only current willingness to continue working in one's organization but also the future intention to work beyond retirement age among older adults. This supports the reasoning that stereotype threat, through increasing stress, can lead to behavioral disidentification – namely, the desire to withdraw from work as soon as possible. Moreover, since the relationship between stereotype threat and perceived stress was moderate, the authors conclude that stereotype threat, although related to stress, is a relatively orthogonal construct.

Interestingly, the authors' results indicated that the number of diseases reported by employees, entered into the model as a control variable, was positively related to stereotype threat. This finding may open significant discussion about the sources of different types of stereotype threat in the workplace and intersectionality of these various stereotype threats. Older employees may suffer from stereotype threat related to age, but also from threats related to chronic illness (as illness is stereotypically attributed to older adults), and older adult women may experience gender-related stereotype threat. The question arises whether the impact of different stereotype threats is relatively independent or more cumulative. Although this issue has been identified in the literature on stereotype threat [28], the research remains limited. The only study that examined this hypothesis indicated that the level of age-related stereotype threat is equivalent among male and female workers, suggesting that different stereotype threats may be independent [18].

Additionally, age-related stereotype threat in the workplace does not only impact older adults, but is also observed in younger adults. The question arises, whether the mechanism of stereotype threat is the same across these 2 age groups. The pattern of findings in research exploring consequences of daily stereotype threat events in these 2 groups suggests that some stereotype outcomes are age specific. Therefore, the mechanism might be partially different, as older adults appear more prone to rumination [16] and vulnerable to disengagement under stereotype threat. Additional research should address this issue.

### Study limitations and future directions

Several limitations of this study should be noted. First, the descriptive statistics suggest that the level of stereotype threat in the authors' sample of older adults is relatively low. However, the variability of the stereotype threat items is substantial, suggesting that there were individual differences among participants in the level of experienced stereotype threat. This variability should be further explained in future studies.

Second, the use of a heterogeneous, non-random sample limits the generalizability of the findings, and the authors acknowledge that contextual factors such as organizational culture, sector, or interpersonal dynamics may influence the salience of stereotype threat and stress. Future studies should examine how such environmental variables moderate these relationships, particularly in samples with higher reported levels of stereotype threat.

Third, the authors did not include important moderators of stereotype threat, such as group identification, endorsement of age-related stereotypes, or stigma-consciousness [29]. All of these factors have been shown to be significant moderators of laboratory-induced stereotype threat in older participants, and should be further tested in applied settings.

Fourth, the authors' study implemented self-report measures of stereotype threat, stress and willingness to continue work beyond retirement age. As recommended by Podsakoff et al. [30] common method bias related to self-report measures was controlled through temporal separation of scale measurement, protecting respondent anonymity by using different scale endpoints. However, more elaborated methods to control this bias should be implemented in the future research. Fifth, the major limitation of the study lies in a cross-sectional design employed. To establish a more plausible cause-effect relationship, temporal sequencing between variables is needed, which is possible through longitudinal studies.

Sixth, measuring the intention to work beyond the retirement age with a single item constitutes a limitation of this study, as it may not fully capture the complexity of retirement-related decision-making and therefore the results should be interpreted with caution. Nevertheless, according to the theory of planned behavior [31], intention is the most proximal predictor of behavior, shaped by attitudes, subjective norms, and perceived behavioral control. Prior research shows that retirement intentions are strongly associated with actual retirement behavior [32], and single-item measures are widely applied in aging and retirement studies [33]. While such measures may have drawbacks regarding psychometric properties, recent evidence indicates that many single-item measures demonstrate can demonstrate satisfactory validity and test-retest reliability [34,35], e.g., when the construct is narrow in scope [35] or when expected associations are moderate [36]. However, future research should extend this measure by incorporating attitudes, subjective norms, and particularly perceived behavioral control, which may moderate the relationship between intention and behavior [37].

Moreover, future studies should include broader outcome variables, such as willingness to learn and develop skills, job satisfaction, and work-related health. It is plausible that similar mechanisms involving stress may also shape these outcomes in older employees. An important question also arises about the reciprocal relationships between stereotype threat and stress at work – it is possible that more stressful workplaces evoke greater stereotype threat concerns in older participants. Understanding the temporal aspect of stereotype threat and stress relationships is of great theoretical and practical importance. Future studies should address these questions and limitations.

### Practical implications

Findings from this study can be applied in organizational settings through the implementation of interventions, training programs, human resource management practices, and policies aimed at minimizing stereotype threat at work and promoting mental health among aging workers. Raising awareness of the stereotype threat phenomenon and its negative consequences for employees' mental health and retirement intentions among human resource specialists, managers and employees could help combat the stereotypes and, consequently, reduce its prevalence and negative outcomes at individual, organizational, and societal levels.

## CONCLUSIONS

– Retaining older employees and extending their working lives are essential for workforce sustainability in an aging society.
– As the number of older workers grows, it becomes increasingly important to understand the factors influencing their work satisfaction and willingness to remain professionally active beyond retirement age.
– The authors' findings indicate that stereotype threat at work is associated with increased stress levels, which in turn are related to lower intentions to continue working beyond retirement age.
– These results highlight the importance of recognizing the psychological mechanisms – such as stereotype threat and stress – that can undermine older employees' motivation and mental well-being.
– A better understanding of these processes can inform the development of interventions and workplace practices aimed at reducing stereotype threat, supporting mental health, and encouraging the retention of older workers.
